# Risk factors for anastomotic complications following thoracoscopic repair of type III esophageal atresia in neonates: a single-center retrospective cohort study

**DOI:** 10.3389/fped.2026.1743040

**Published:** 2026-02-17

**Authors:** Ziwei Zhang, Like Yuan, Meng Li, Xiaochun Zhu, Wuping Ge, Jialiang Zhou, Lu Xu, Song Tian, Yuanlong Fang, Rong Huang, Xinyue Li, Yasi Wang, Shangjie Xiao

**Affiliations:** 1 Department of Neonatal Surgery, Guangdong Women and Children Hospital, Guangzhou, China; 2 Guangzhou Medical University, Guangzhou, China; 3 Women and Children's Hospital, Southern University of Science and Technology, Guangzhou, China

**Keywords:** anastomotic complications, esophageal atresia, neonatal minimally invasive surgery, operative efficiency, precision surgery, respiratory malformations, surgical learning curve, thoracoscopic repair

## Abstract

**Background:**

Although thoracoscopic repair in China has emerged as a preferred and increasingly adopted minimally invasive approach for esophageal atresia (EA), anastomotic complications remain a major concern, with reported incidences ranging from 15% to 80%. However, the independent risk factors for these complications in a homogeneous surgical population remain poorly characterized.

**Methods:**

This single—center retrospective cohort study recruited neonates with type III EA who underwent primary thoracoscopic repair. Comprehensive demographic, operative, and anatomic variables were gathered. Univariate and multivariable logistic regression analyses were utilized to identify independent predictors for anastomotic leak (AL), anastomotic stricture (AS), and recurrent tracheoesophageal fistula (recurrent TEF).

**Results:**

The cohort had a mean birth weight of 2,597 g and a median operative time of 2.3 h. The incidences of AL, AS, and recurrent TEF were 15.4%, 74.6%, and 6.5%, respectively. Multivariable analysis identified distinct etiological mechanisms: an operative time of ≤2 h independently protected against AL (OR 0.24, 95% CI 0.09–0.63); the institutional learning curve attenuated traditional AS risk factors such as low birth weight; and the presence of a respiratory anomaly was the strongest predictor of recurrent TEF (OR = 8.84; 95% CI, 2.09–33.47). Minimal co-occurrence among the three complications (phi coefficients ≈ 0) confirmed their etiological independence.

**Conclusion:**

The three major anastomotic complications of thoracoscopic EA repair stem from distinct mechanisms. Anastomotic leak was independently protected by operative time ≤2 h (OR 0.24, 95% CI 0.09–0.63). The institutional learning curve attenuated traditional risk factors for anastomotic stricture (incidence 74.6%). Recurrent TEF was predominantly associated with respiratory anomalies (OR 8.84, 95% CI 2.09–33.47). This highlights the need for precision-based management. The robustness of these key associations was further confirmed through sensitivity analyses and enhanced methodological rigor, including assessments of predictor correlations and model diagnostics.

## 1 Introduction

Esophageal atresia (EA) is a life-threatening congenital anomaly that necessitates urgent surgical correction in neonates. Gross type C (type III) EA, characterized by a proximal blind pouch and a distal tracheoesophageal fistula, represents the predominant subtype, accounting for over 85% of all cases (1, 2). Its high prevalence, coupled with the feasibility of primary anastomosis, has made it a central focus for surgical outcomes research. The primary challenge lies in achieving a tension-free and well-vascularized anastomosis in neonates, whose fragile thoracic anatomy and immature physiology render them particularly susceptible to postoperative complications (3).

The introduction of thoracoscopic repair (TR) has significantly advanced the management of EA by providing a minimally invasive approach that reduces musculoskeletal sequelae and enhances visualization within the confined neonatal thoracic cavity (4, 5). Despite these technical advantages, anastomotic complications–specifically anastomotic leak (AL), anastomotic stricture (AS), and recurrent tracheoesophageal fistula (recurrent TEF)–remain major determinants of postoperative morbidity, long-term functional outcomes, and prolonged hospitalization (3, 6). These complications continue to pose a significant barrier to optimizing recovery and achieving consistent surgical success.

Although numerous studies have demonstrated the non-inferiority of TR compared with open repair, important knowledge gaps persist (3). Many existing studies are either descriptive or rely on univariate analyses that cannot adjust for confounding variables. Furthermore, research often combines various EA subtypes and surgical techniques, thereby obscuring procedure-specific risk factors. Consequently, the independent predictors uniquely associated with AL, AS, and recurrent TEF following thoracoscopic repair for type III EA specifically are poorly defined. It also remains uncertain whether these complications share common underlying mechanisms or arise from distinct etiological pathways (3, 7).

Therefore, this study aimed to dissect the distinct etiologies of major anastomotic complications by employing multivariable analysis in a homogeneous cohort of neonates with type III EA undergoing primary TR. We sought to determine whether the risk of each complication is primarily influenced by modifiable technical factors or by inherent patient-related characteristics, and to clarify the potential interrelationship among these complications.

## 2 Materials and methods

### 2.1 Study design and patient population

This single-center retrospective cohort study was conducted in accordance with the Declaration of Helsinki and was approved by the Medical Ethics Committee of Guangdong Women and Children Hospital (Approval No.: 20251102). The requirement for informed consent was waived due to the retrospective nature of the study, in compliance with national regulations and institutional policies.

All neonates with gross type III esophageal atresia (EA) who underwent thoracoscopic repair at our institution between January 2017 and June 2025 were consecutively screened for eligibility. The following exclusion criteria were applied: 1) non—neonatal surgery (operation performed beyond 28 days of life); 2) patients who underwent only palliative procedures (fistula ligation and/or gastrostomy without anastomosis); 3) postoperative mortality and discharge against medical advice before outcome assessment.

### 2.2 Data collection and preprocessing

Comprehensive demographic, preoperative, intraoperative, and postoperative data were extracted from electronic medical records. The collected variables and their definitions are as follows:

**Demographics:** Sex, gestational age (weeks), birth weight (grams), and age at surgery (days).

**Operative Variables:**

*Operative Time: From skin incision to closure, categorized as Short (≤2 h), Medium (2–3 h), or Long (>3 h).

*Operative Complexity: A center-defined classification reflecting the performance of concomitant procedures during the primary thoracoscopic repair. This categorization reflects that concomitant procedures, while not increasing the intrinsic technical difficulty of the esophageal anastomosis, prolong operative time, increase tissue manipulation, and heighten physiological stress. These factors may collectively influence the perioperative environment and anastomotic healing.

C0 (Basic complexity): Isolated thoracoscopic repair of TEF and esophageal anastomosis.

C1 (Moderate complexity): C0 procedure plus one additional procedure: either a laparoscopic upper gastrointestinal procedure, a simple feeding/decompression stoma, or a single non-major superficial procedure.

C2 (High complexity): C0 procedure plus any of the following: a colostomy, a complex reconstruction of the distal gastrointestinal tract, or two or more additional procedures.

*Surgeon: The primary operating surgeon (one of two senior pediatric surgeons).

*One-Lung Ventilation: Recorded as “Yes” or “No” based on intraoperative anesthesia records.

Anatomic Variables (3)

*Proximal Pouch Level: Determined intraoperatively by referencing the thoracic vertebral column and categorized as: High (pouch tip at or cranial to the T2 vertebra), Medium (between T2 and T3 vertebrae), or Low (at or caudal to the T3 vertebra).

*Gap Length: Measured preoperatively on CT esophagogram (Preoperative_CT_Gap_Length) and directly during surgery (Intraoperative_Gap_Length) in centimeters.

*Tension Index (3, 8): A dimensionless ratio calculated as Intraoperative_Gap_Length (cm)/Birth Weight (kg), as previously described (8) to quantitatively assess the relative tension at the anastomotic site.

Associated Anomalies (3): Associated congenital anomalies were systematically documented as present or absent for the following systems based on objective diagnostic criteria:

*Cardiovascular: Major cardiac anomalies requiring surgical intervention or long-term follow-up (e.g., septal defects, tetralogy of Fallot, patent ductus arteriosus), diagnosed via echocardiography.

*Respiratory: The presence of a respiratory anomaly was strictly defined based on objective preoperative or intraoperative findings, confirmed by laryngotracheobronchoscopy and/or cross-sectional imaging, and included any of the following:

Laryngotracheomalacia, defined as >70% expiratory collapse of the airway lumen on dynamic bronchoscopy.

Vocal cord paralysis, documented by laryngoscopy or bronchoscopy.

Laryngeal cleft, diagnosed by direct laryngoscopy or bronchoscopy.

Subglottic stenosis, confirmed by bronchoscopy.

Tracheal bronchus or other major airway anomalies, identified by bronchoscopy or computed tomography (CT).

Congenital pulmonary airway malformation (CPAM) or bronchopulmonary sequestration (BPS), diagnosed by prenatal or postnatal CT imaging.

Isolated, mild laryngomalacia without significant functional impairment was excluded from this category.

*Gastrointestinal anomalies: Documented by radiography, ultrasonography, or operative findings (e.g., anorectal malformations, duodenal atresia, intestinal malrotation).

*Genitourinary anomalies: Identified by renal ultrasonography (e.g., renal agenesis/dysplasia, hydronephrosis, vesicoureteral reflux).

*Musculoskeletal anomalies: Confirmed by radiography or physical examination (e.g., vertebral anomalies, limb defects, rib abnormalities).

The variable Any_Anomaly was defined as the presence of one or more major congenital anomalies in any of the aforementioned systems.

Categorical variables (e.g., sex, gestational age category, birth weight category, operative time category, operation complexity, surgeon, proximal pouch level, use of one-lung ventilation, and the presence of associated anomalies) were converted into factors for analysis. The primary outcomes (anastomotic leak, stricture, recurrent TEF) were transformed into binary factor variables (“Yes” “No”).

### 2.3 Outcome definitions

The primary outcomes were the occurrence of three major anastomotic-related complications, defined as follows (3, 9):

**Anastomotic Leak:** A clinical diagnosis was made based on one or more of the following criteria: (a) clinical evidence of persistent salivary or food drainage from the surgical incision or chest tube; (b) radiological evidence of contrast extravasation on esophagogram; (c) suggestive radiological findings (e.g., new-onset pneumomediastinum or pleural effusion) in the appropriate clinical context.

**Anastomotic Stricture:** A clinical diagnosis was made based on one or more of the following criteria: (a) direct endoscopic management of food bolus impaction; (b) presence of feeding difficulties AND objective radiological confirmation; (c) detection of significant luminal narrowing on routine postoperative surveillance esophagogram.

**Recurrent Tracheoesophageal Fistula:** Diagnosed by bronchoscopy with methylene blue test confirmation.

### 2.4 Surgical technique

All procedures were performed by one of two senior pediatric surgeons using a standardized thoracoscopic approach under CO_2_ pneumothorax. The fundamental steps included fistula division, meticulous mobilization of the proximal and distal esophageal pouches, and tension-free single-layer anastomosis (5, 10, 11).

### 2.5 Statistical analysis

**Study Design and Aim:** This retrospective cohort study aimed to identify factors independently associated with anastomotic complications, not to establish causal relationships.

**Univariate Screening**: To identify candidate variables for multivariable modeling, we first performed univariate comparisons between patients with and without each complication. Continuous variables were compared using Welch Two Sample t-test or the Wilcoxon rank-sum test, as appropriate based on data distribution. Categorical variables were compared using Pearson's Chi-squared test or Fisher's exact test, as appropriate. Variables with a *p*-value < 0.1 in these univariate comparisons were selected as candidates for the subsequent multivariable logistic regression model. The complete results of these univariate comparisons are detailed in Supplementary Tables S1–S3.

**Validation of Screening Method and Formal Variable Selection**: To ensure methodological consistency with the final regression model and to validate the candidate variable set, we performed univariate logistic regression for all predictors. The set of candidate variables (*p* < 0.1) identified by this method was consistent with the initial screening approach. The complete results of these univariate logistic regression analyses are provided in Supplementary Table S5.

**Assessment of Predictor Correlation**: Prior to multivariable modeling, we assessed potential multicollinearity among the candidate continuous predictors by calculating Pearson’s correlation coefficients. No pair of variables showed a correlation coefficient with an absolute value greater than 0.7, indicating that multicollinearity would not preclude their simultaneous inclusion in a regression model (Supplementary Table S6 and Supplementary Figure S1).

**Multivariable Analysis**: Candidate predictors for each complication were entered into separate multivariable logistic regression models to identify independent risk factors. Results are presented as odds ratios (OR) with 95% confidence intervals (CI). A two-tailed *p*-value < 0.05 was considered statistically significant.

**Regression Diagnostics:** To validate the robustness and goodness-of-fit of the final multivariable models, we performed comprehensive regression diagnostics. This included calculating the Variance Inflation Factor (VIF) to further assess multicollinearity and the Hosmer-Lemeshow test. The results confirmed the absence of critical multicollinearity (all VIFs < 2.5) and adequate model fit (Supplementary Table S7).

Model-Specific Predictor Treatment: The treatment of predictors (continuous vs. categorical) was determined by clinical interpretability and the specific research question for each outcome. For anastomotic leak (AL), operative time and birth weight were modeled using clinically established categories (e.g., ≤2 h vs. >2 h; birth weight categories) to provide actionable risk thresholds. For anastomotic stricture (AS), birth weight and the tension index were retained as continuous variables to explore potential linear dose-response relationships and maximize statistical power. This tailored approach ensures that the model for each complication is both statistically appropriate and clinically meaningful. To test the robustness of this approach, we performed sensitivity analyses treating these variables in their alternate forms (e.g., operative time as continuous). The consistency of findings across these analyses is summarized in Supplementary Table S4.

Correlation and Co-occurrence: Correlation between continuous variables was assessed using Pearson's correlation coefficient. Co-occurrence of complications was visualized using a Venn diagram and the strength of association was quantified using the Phi coefficient for binary variables.

All analyses were performed using R software (version 4.4.3; R Foundation for Statistical Computing, Vienna, Austria). Primary packages included: stats (for core regression), car (for VIF calculation), ResourceSelection (for Hosmer-Lemeshow test), and tidyverse (for data management and visualization).

## 3 Results

### 3.1 Patient cohort

**Figure 1:**
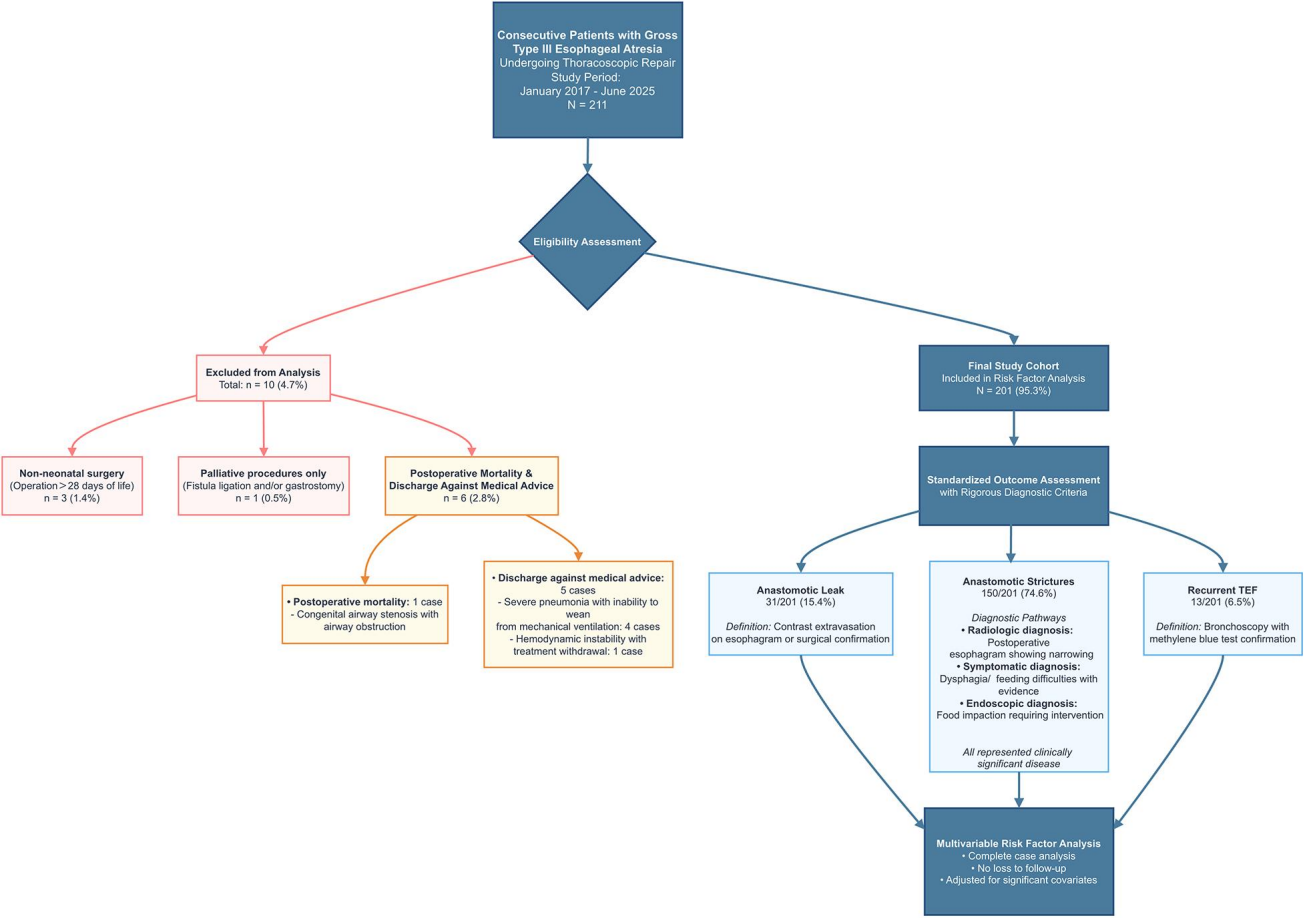
Flowchart of patient selection for the study cohort.

A total of 201 neonates with type III EA who underwent thoracoscopic repair between January 2017 and June 2025 comprised the final study cohort (Figure 1).

Baseline demographic and clinical characteristics are summarized in Table 1.

**Table 1:** Baseline characteristics of the study cohort (*N* = 201): neonates with type III esophageal atresia undergoing primary thoracoscopic repair.

Variable	*N* = 201^a^
Age at Surgery (days)	5.35 (3.42)
Gestational Age (weeks)	37.91 (2.46)
Birth Weight (g)	2,597.49 (566.02)
Operative Time (hours)	2.34 (0.80)
One Lung Ventilation	90 (44.8%)
Proximal Pouch Level	
High (T1-T2)	82 (40.8%)
Low (T3-T4)	28 (13.9%)
Medium (T2-T3)	91 (45.3%)
Preoperative Gap Length (cm)	1.29 (0.74)
Intraoperative Gap Length (cm)	1.81 (0.74)
Tension Index	0.74 (0.39)
Sex	
Female	82 (40.8%)
Male	119 (59.2%)
Gestational Age Category	
Post-term (≥42 weeks)	1 (0.5%)
Preterm (<37 weeks)	54 (26.9%)
Term (37–41 weeks)	146 (72.6%)
Birth Weight Category	
Extremely low BW (<1,500 g)	4 (2.0%)
Low BW (1,500–2,499 g)	71 (35.3%)
Normal BW (≥2,500 g)	126 (62.7%)
Operative Time Category	
Long (>3 h)	33 (16.4%)
Medium (2–3 h)	67 (33.3%)
Short (≤2 h)	101 (50.2%)
Operation Complexity	
Basic complexity	186 (92.5%)
High complexity	8 (4.0%)
Moderate complexity	7 (3.5%)
Cardiovascular Anomalies	22 (10.9%)
Respiratory Anomalies	13 (6.5%)
Genitourinary Anomalies	10 (5.0%)
Gastrointestinal Anomalies	17 (8.5%)
Musculoskeletal Anomalies	36 (17.9%)
Any Anomaly	74 (36.8%)

^a^ Mean (SD); *n* (%).

The mean gestational age was 37.91 ± 2.46 weeks, and the mean birth weight was 2,597.49 ± 566.02 grams. The majority of procedures (50.2%) were completed within 2 h (Short operative time category). Associated anomalies were present in 36.8% of patients.

### 3.2 Incidence of complications

The overall incidences of major anastomotic complications were as follows: AL in 15.4% (31/201), AS in 74.6% (150/201), and recurrent TEF in 6.5% (13/201). The univariate analyses comparing patient characteristics by complication status are detailed in Table 2 (anastomotic leak), Table 3 (anastomotic stricture) and Table 4 (recurrent TEF).

**Table 2:** Comparison of patient characteristics by anastomotic leak Status following thoracoscopic repair.

Variable	0 *N* = 170^a^	1 *N* = 31^a^	*p*-value^b^
Age at Surgery (days)	5.19 (3.28)	6.26 (4.02)	0.10
Gestational Age (weeks)	37.81 (2.50)	38.49 (2.13)	0.2
Birth Weight (g)	2,574.56 (572.80)	2,723.23 (517.95)	0.2
Operative Time (hours)	2.25 (0.71)	2.80 (1.08)	0.004
One Lung Ventilation	79 (46.5%)	11 (35.5%)	0.3
Proximal Pouch Level			0.8
High (T1-T2)	69 (40.6%)	13 (41.9%)	
Low (T3-T4)	25 (14.7%)	3 (9.7%)	
Medium (T2-T3)	76 (44.7%)	15 (48.4%)	
Preoperative Gap Length (cm)	1.28 (0.71)	1.33 (0.87)	>0.9
Intraoperative Gap Length (cm)	1.80 (0.72)	1.82 (0.85)	0.8
Tension Index	0.75 (0.40)	0.70 (0.37)	0.4
Sex			0.3
Female	72 (42.4%)	10 (32.3%)	
Male	98 (57.6%)	21 (67.7%)	
Gestational Age Category			0.10
Post-term (≥42 weeks)	0 (0.0%)	1 (3.2%)	
Preterm (<37 weeks)	48 (28.2%)	6 (19.4%)	
Term (37–41 weeks)	122 (71.8%)	24 (77.4%)	
Birth Weight Category			0.037
Extremely low BW (<1,500 g)	3 (1.8%)	1 (3.2%)	
Low BW (1,500–2,499 g)	66 (38.8%)	5 (16.1%)	
Normal BW (≥2,500 g)	101 (59.4%)	25 (80.6%)	
Operative Time Category			0.001
Long (>3 h)	21 (12.4%)	12 (38.7%)	
Medium (2–3 h)	59 (34.7%)	8 (25.8%)	
Short (≤2 h)	90 (52.9%)	11 (35.5%)	
Operation Complexity			0.052
Basic complexity	160 (94.1%)	26 (83.9%)	
High complexity	6 (3.5%)	2 (6.5%)	
Moderate complexity	4 (2.4%)	3 (9.7%)	
Cardiovascular Anomalies	18 (10.6%)	4 (12.9%)	0.8
Respiratory Anomalies	11 (6.5%)	2 (6.5%)	>0.9
Genitourinary Anomalies	9 (5.3%)	1 (3.2%)	>0.9
Gastrointestinal Anomalies	14 (8.2%)	3 (9.7%)	0.7
Musculoskeletal Anomalies	32 (18.8%)	4 (12.9%)	0.4
Any Anomaly	64 (37.6%)	10 (32.3%)	0.6

^a^ Mean (SD); *n* (%).

^b^ Wilcoxon rank sum test; Pearson's Chi-squared test; Fisher's exact test.

**Table 3:** Comparison of patient characteristics by anastomotic stricture Status following thoracoscopic repair.

Variable	0 *N* = 51^a^	1 *N* = 150^a^	*p*-value^b^
Age at Surgery (days)	4.84 (2.73)	5.53 (3.61)	0.2
Gestational Age (weeks)	37.27 (2.99)	38.13 (2.21)	0.2
Birth Weight (g)	2,366.08 (658.67)	2,676.17 (509.80)	0.003
Operative Time (hours)	2.39 (0.78)	2.32 (0.81)	0.3
One Lung Ventilation	25 (49.0%)	65 (43.3%)	0.5
Proximal Pouch Level			0.3
High (T1-T2)	23 (45.1%)	59 (39.3%)	
Low (T3-T4)	4 (7.8%)	24 (16.0%)	
Medium (T2-T3)	24 (47.1%)	67 (44.7%)	
Preoperative Gap Length (cm)	1.36 (0.76)	1.27 (0.73)	0.4
Intraoperative Gap Length (cm)	1.86 (0.77)	1.79 (0.73)	0.4
Tension Index	0.86 (0.44)	0.70 (0.37)	0.012
Sex			0.6
Female	19 (37.3%)	63 (42.0%)	
Male	32 (62.7%)	87 (58.0%)	
Gestational Age Category			0.3
Post-term (≥42 weeks)	0 (0.0%)	1 (0.7%)	
Preterm (<37 weeks)	18 (35.3%)	36 (24.0%)	
Term (37–41 weeks)	33 (64.7%)	113 (75.3%)	
Birth Weight Category			0.002
Extremely low BW (<1,500 g)	3 (5.9%)	1 (0.7%)	
Low BW (1,500–2,499 g)	25 (49.0%)	46 (30.7%)	
Normal BW (≥2,500 g)	23 (45.1%)	103 (68.7%)	
Operative Time Category			0.9
Long (>3 h)	9 (17.6%)	24 (16.0%)	
Medium (2–3 h)	18 (35.3%)	49 (32.7%)	
Short (≤2 h)	24 (47.1%)	77 (51.3%)	
Operation Complexity			0.5
Basic complexity	46 (90.2%)	140 (93.3%)	
High complexity	2 (3.9%)	6 (4.0%)	
Moderate complexity	3 (5.9%)	4 (2.7%)	
Cardiovascular Anomalies	9 (17.6%)	13 (8.7%)	0.076
Respiratory Anomalies	4 (7.8%)	9 (6.0%)	0.7
Genitourinary Anomalies	3 (5.9%)	7 (4.7%)	0.7
Gastrointestinal Anomalies	6 (11.8%)	11 (7.3%)	0.4
Musculoskeletal Anomalies	6 (11.8%)	30 (20.0%)	0.2
Any Anomaly	22 (43.1%)	52 (34.7%)	0.3

^a^ Mean (SD); *n* (%).

^b^ Wilcoxon rank sum test; Pearson's Chi-squared test; Fisher's exact test.

**Table 4:** Comparison of patient characteristics by recurrent tracheoesophageal Fistula Status following thoracoscopic repair.

Variable	0 *N* = 188^a^	1 *N* = 13^a^	*p*-value^b^
Age at Surgery (days)	5.35 (3.46)	5.46 (2.85)	0.9
Gestational Age (weeks)	37.89 (2.51)	38.24 (1.57)	>0.9
Birth Weight (g)	2,592.47 (573.03)	2,670.00 (465.47)	0.9
Operative Time (hours)	2.33 (0.81)	2.43 (0.68)	0.4
One Lung Ventilation	85 (45.2%)	5 (38.5%)	0.6
Proximal Pouch Level			0.6
High (T1-T2)	78 (41.5%)	4 (30.8%)	
Low (T3-T4)	27 (14.4%)	1 (7.7%)	
Medium (T2-T3)	83 (44.1%)	8 (61.5%)	
Preoperative Gap Length (cm)	1.29 (0.75)	1.23 (0.56)	>0.9
Intraoperative Gap Length (cm)	1.81 (0.72)	1.79 (1.05)	0.5
Tension Index	0.75 (0.39)	0.73 (0.50)	0.6
Sex			0.12
Female	74 (39.4%)	8 (61.5%)	
Male	114 (60.6%)	5 (38.5%)	
Gestational Age Category			>0.9
Post-term (≥42 weeks)	1 (0.5%)	0 (0.0%)	
Preterm (<37 weeks)	51 (27.1%)	3 (23.1%)	
Term (37–41 weeks)	136 (72.3%)	10 (76.9%)	
Birth Weight Category			0.5
Extremely low BW (<1,500 g)	4 (2.1%)	0 (0.0%)	
Low BW (1,500–2,499 g)	68 (36.2%)	3 (23.1%)	
Normal BW (≥2,500 g)	116 (61.7%)	10 (76.9%)	
Operative Time Category			0.6
Long (>3 h)	30 (16.0%)	3 (23.1%)	
Medium (2–3 h)	62 (33.0%)	5 (38.5%)	
Short (≤2 h)	96 (51.1%)	5 (38.5%)	
Operation Complexity			>0.9
Basic complexity	173 (92.0%)	13 (100.0%)	
High complexity	8 (4.3%)	0 (0.0%)	
Moderate complexity	7 (3.7%)	0 (0.0%)	
Cardiovascular Anomalies	21 (11.2%)	1 (7.7%)	>0.9
Respiratory Anomalies	9 (4.8%)	4 (30.8%)	0.006
Genitourinary Anomalies	10 (5.3%)	0 (0.0%)	>0.9
Gastrointestinal Anomalies	17 (9.0%)	0 (0.0%)	0.6
Musculoskeletal Anomalies	34 (18.1%)	2 (15.4%)	>0.9
Any Anomaly	69 (36.7%)	5 (38.5%)	>0.9

^a^ Mean (SD); *n* (%).

^b^ Wilcoxon rank sum test; Pearson's Chi-squared test; Fisher's exact test.

### 3.3 Univariate analysis

Univariate comparisons (Welch t-test for continuous variables; Chi-squared or Fisher's exact test for categorical variables) between patients with and without each complication are detailed in Supplementary Tables S1–S3. For AL, shorter operative time (*p* *=* *0.001*) and higher birth weight category (*p* = 0.037) were associated with lower risk. For AS, lower birth weight (*p* = 0.003) and a higher tension index *(p* = 0.012) were significant risk factors. For recurrent TEF, the presence of respiratory anomalies was the most significant association (30.8% vs. 4.8%, *p* = 0.006). Variables with a *p*-value < 0.1 in these univariate analyses were selected as candidates for multivariable regression. To validate this screening method and ensure methodological consistency, we also performed univariate logistic regression for all predictors (Supplementary Table S5). The candidate variable sets were consistent.

### 3.4 Multivariable regression analysis

**Figure 2:**
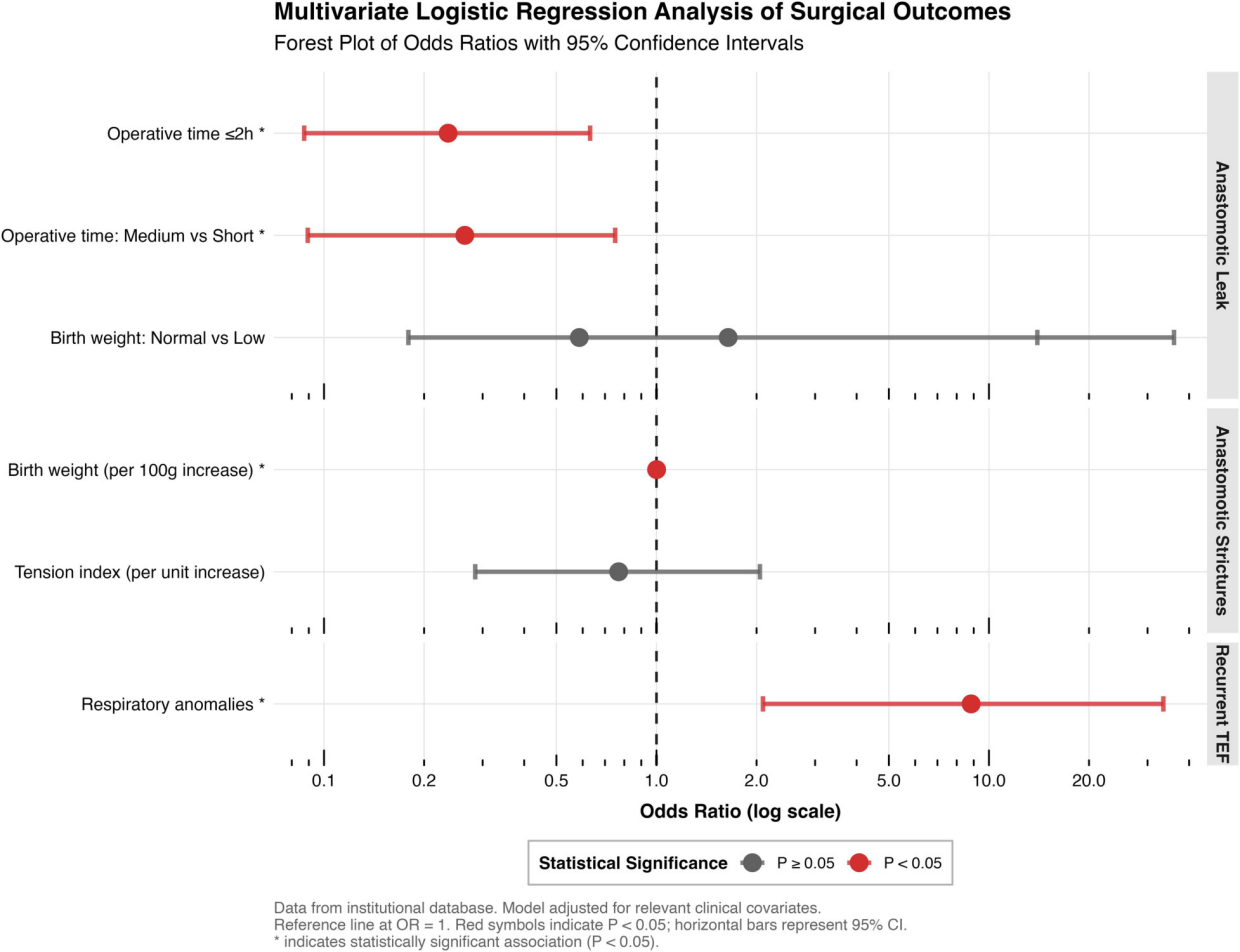
Forest plot of independent risk factors for anastomotic complications identified by multivariable logistic regression analysis.

Prior to building the final multivariable models, we assessed multicollinearity among candidate predictors using correlation matrices (Supplementary Table S6), and no critical correlation (|*r*| > 0.7) was found. Independent predictors identified through multivariable logistic regression are summarized in the forest plot (Figure 2).

For AL, shorter operative time (≤2 h) remained a significant protective factor compared to longer operations (*OR 0.24, 95% CI 0.09–0.63, p* *=* *0.004*). Regression diagnostics confirmed the model's robustness (all VIFs < 1.1; see Supplementary Table S7), and sensitivity analysis treating operative time as a continuous variable confirmed a strong dose-response relationship, with each additional hour of operative time associated with an approximately twofold increase in the odds of leak (*OR* *=* *2.17, 95% CI 1.40–3.45, p* *<* *0.001*; see Supplementary Table S4a). Operative time categorized as “Medium” (2–3 h) also showed a protective effect vs. “Long” (*OR 0.27, 95% CI 0.09–0.75, p* *=* *0.014*). Notably, several factors significant in univariate analyses—including the use of one-lung ventilation *(p* = 0.037), higher birth weight category (*p* = 0.050), and increased operative complexity (*p* = 0.086)—failed to retain independent significance in the multivariable model, suggesting their effects may be mediated through operative time.

**Figure 4:**
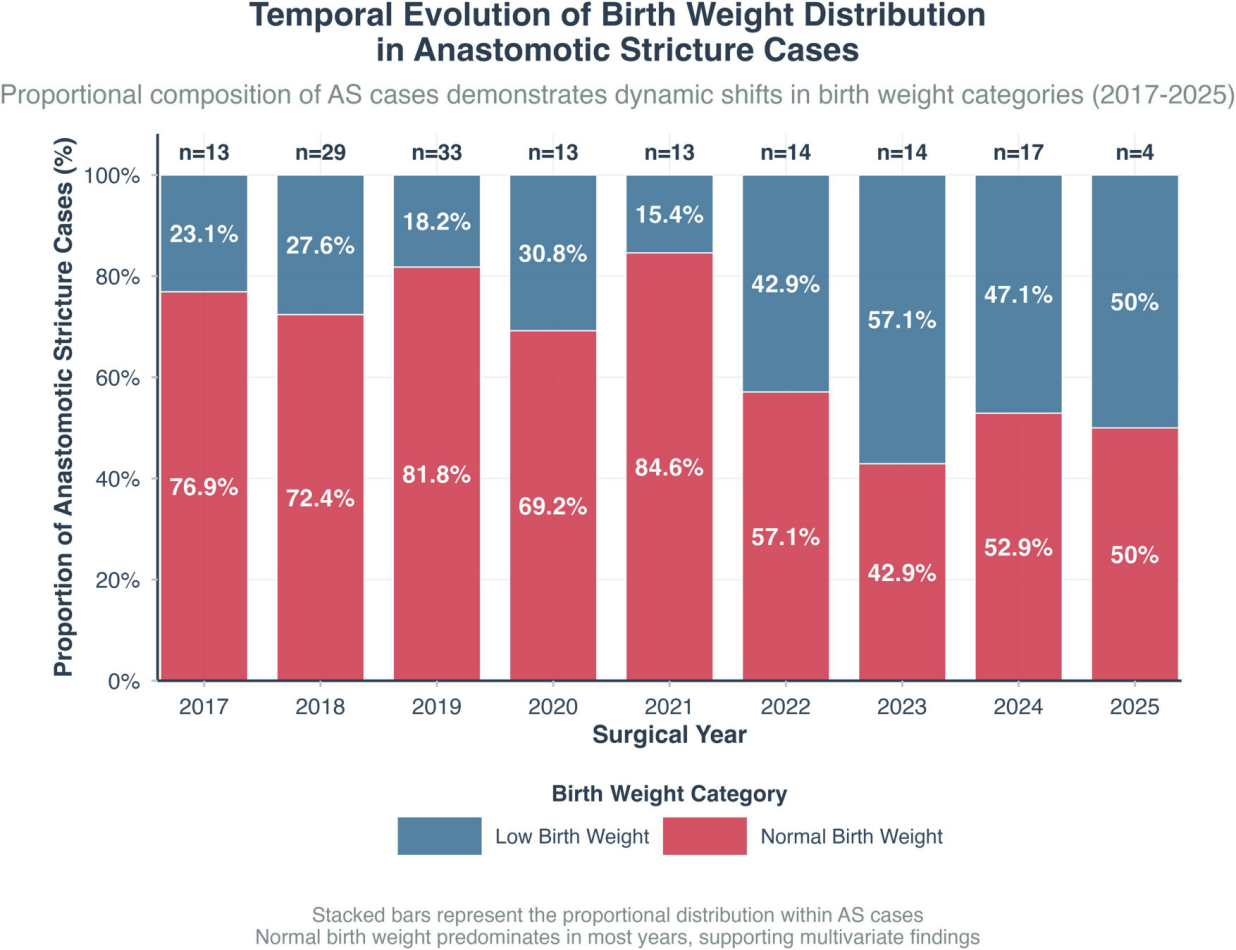
Longitudinal trends in patient birth weight categories, institutional case volume, and anastomotic stricture rate over the study period.

For AS, the multivariable model revealed a distinct pattern after adjustment for confounders including gestational age. When analyzed as a continuous variable, each 100-gram increase in birth weight was associated with a statistically significant but clinically negligible increase in risk (*OR 1.001, 95% CI 1.000–1.002, p* *=* *0.019*). This model result suggests that after accounting for other factors (e.g., the institutional learning curve reflected in surgical proficiency over time), birth weight *per se* had no meaningful independent effect on stricture risk. The significant association between lower birth weight and AS observed in univariate analysis (*p* *=* *0.003)* therefore likely operated through correlated anatomical and technical challenges (partially captured by variables like the tension index), which were attenuated by surgical experience. Accordingly, the tension index, also analyzed continuously, was not retained as an independent predictor (OR 0.77, 95% CI 0.28–2.05, *p* = 0.600). Anatomical factors such as proximal pouch level and gap measurements were also not independently significant. Figure 4 depicts the longitudinal distribution of strictures and their relationship to patient characteristics. The final model for AS, along with its diagnostic checks indicating no critical multicollinearity (VIFs < 2.5) and acceptable model fit, is detailed in Supplementary Table S7.

For recurrent TEF, the presence of a respiratory anomaly was the strongest independent risk factor (*OR 8.84, 95% CI 2.09–33.47, p* *=* *0.002*). This factor was retained for multivariable analysis based on strong clinical plausibility and its significant univariate association in comparative tests (30.8% vs. 4.8%, *p* *=* *0.006*; Table 4), despite its *p*-value in the formal univariate logistic regression screening exceeding our pre-specified threshold—a nuance likely influenced by the low event rate. Other anomalies significant in univariate analyses were not independent predictors.

Detailed outputs of all multivariable models, including sensitivity analyses, are provided in Supplementary Table S4.

### 3.5 Co-occurrence of complications and etiological independence

**Figure 3:**
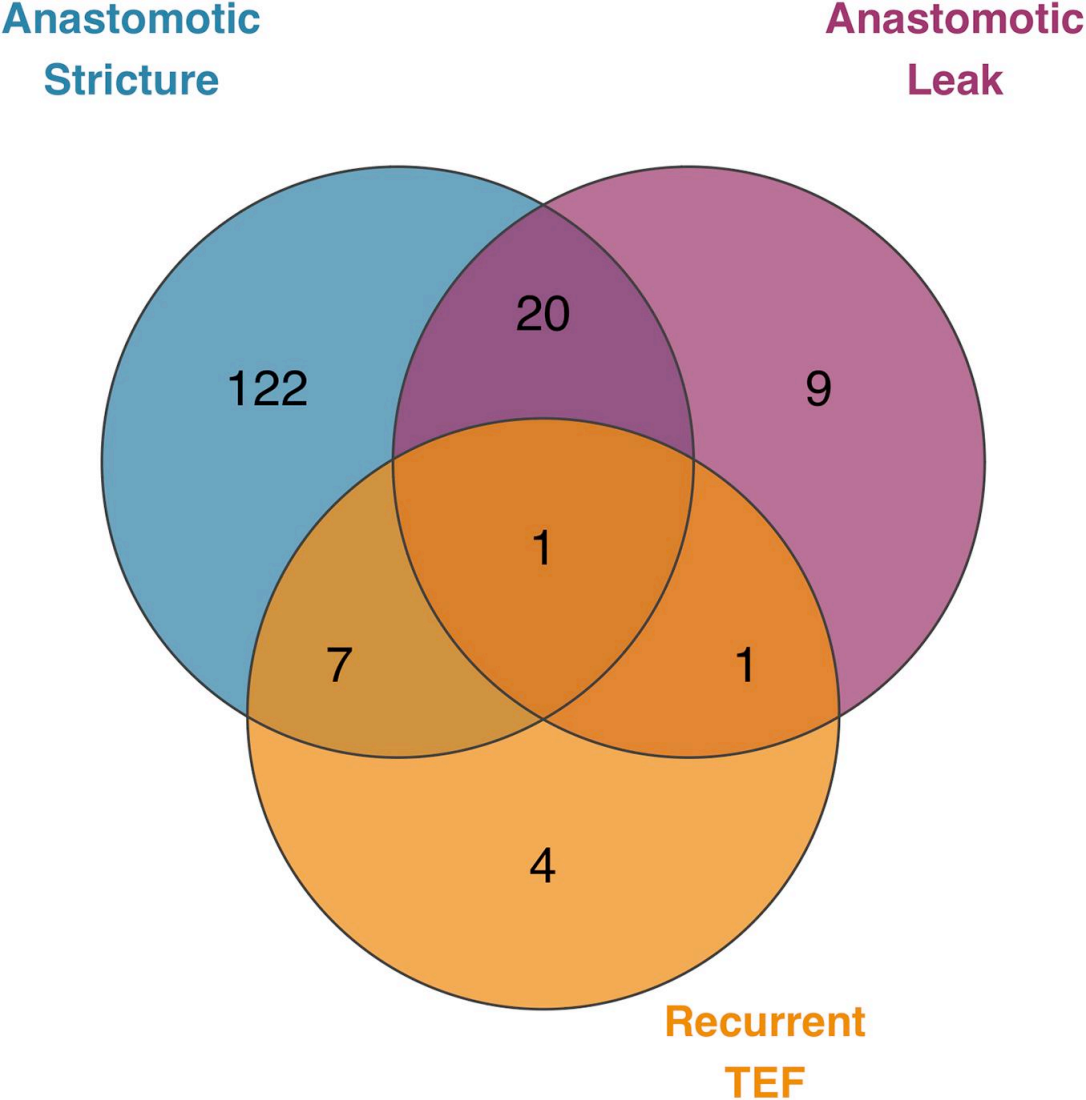
Venn diagram illustrating the overlap Among the three Major anastomotic complications.

Multiple anastomotic complications occurred in 14.4% (29/201) of patients. To visualize the overlap between different complication types, a Venn diagram was constructed (Figure 3).

Correlation analysis based on this distribution revealed negligible associations between individual complications (phi coefficients ≈ 0), providing quantitative support for distinct underlying etiologies.

## 4 Discussion

### 4.1 Principal findings and etiological framework

This large, homogeneous cohort study delineates distinct risk profiles for the three major anastomotic complications following thoracoscopic repair of type III EA. Multivariable analysis identified that AL was independently protected by an operative time of ≤2 h (*OR 0.24, 95% CI 0.09–0.63, p* *=* *0.004*). For AS (incidence 74.6%), the influence of traditional risk factors like low birth weight diminished when accounting for the institutional learning curve. Recurrent TEF was most strongly predicted by the presence of a respiratory anomaly (*OR 8.84, 95% CI 2.09–33.47, p* *=* *0.002*). The striking etiological independence of these complications is powerfully illustrated by their distribution pattern (Figure 3), where the vast majority occurred in isolation (e.g., 74.4% for AS alone, 2.4% for recurrent TEF alone), and co-occurrence was rare (e.g., only 0.6% for AL + recurrent TEF). These findings underscore the necessity of adopting a precision-based, complication-specific preventive strategy rather than a uniform risk mitigation approach (52).

### 4.2 Anastomotic leak: operative efficiency as the cornerstone of safety in thoracoscopy

Our analysis definitively identifies shorter operative time as the foremost independent predictor for reduced AL risk (49, 50). Procedures completed within 2 h were associated with a markedly reduced risk of leakage (OR 0.24). We interpret operative time not as a direct causal factor, but as a surrogate metric for overall procedural efficiency, technical fluency, and adherence to a standardized operative protocol, reflecting the surgical team's capacity to execute key steps with minimal delay and tissue trauma. The robustness of this finding is underscored by sensitivity analysis: when operative time was analyzed as a continuous variable, a clear dose-response relationship emerged, with each hour of increase significantly elevating leak risk (*OR* *=* *2.17, 95% CI 1.40–3.45, p* *<* *0.001*; Supplementary Table S4a). This reinforces that operative time is a reliable composite metric of procedural efficiency and technical mastery in our specific thoracoscopic context. This efficiency is influenced by two key, often intertwined, factors: (1) the inherent anatomical challenge, such as a long gap between esophageal pouches, which necessitates more extensive mobilization and inherently prolongs the procedure; and (2) the surgeon's technical proficiency and stage on the learning curve, where greater fluency translates to faster, more atraumatic execution of each surgical step. This finding refines our understanding of the thoracoscopic approach. While a previous large-scale comparison found no significant difference in AL rates between thoracoscopic and open approaches (OR = 1.92, *p* = 0.062) (4, 5), our study drills down into a pure thoracoscopic cohort to reveal that technical efficiency, quantified by operative time, is a critical modifiable determinant of anastomotic integrity specific to this technique. The observation that other technically-linked variables (e.g., one-lung ventilation time) failed to retain independent significance in the multivariable model suggests that the ultimate determinant of leak risk is the overarching capacity of the surgical team to overcome anatomical challenges efficiently and execute a secure anastomosis with technical mastery (5, 11, 12). Therefore, beyond merely demonstrating non-inferiority, our data provide a procedure-specific optimization target for TR (4, 5, 9, 10).

### 4.3 Anastomotic stricture: the interplay of technique, surveillance, and management evolution

The anastomotic stricture (AS) rate of 74.6% in our cohort must be interpreted within the context of our follow-up protocol and management strategy. Firstly, our definition of AS encompassed not only symptomatic cases but also asymptomatic luminal narrowing identified through routine surveillance esophagograms. Secondly, our proactive, long-term follow-up protocol—specifically, monthly esophagograms during the first six postoperative months, followed by evaluations every six months thereafter—complies with guidelines (13) and results in higher detection rates (51). Our observed high rate aligns with reports in the literature where intensive surveillance is employed. For instance, a single-center study reported that 79% of patients required endoscopic dilatation after repair (14). This substantial burden of stricture management is a well-recognized phenomenon following EA repair, as highlighted in dedicated reviews which emphasize the first two years postoperatively as a critical period for stricture development requiring close monitoring (15). Importantly, all symptomatic strictures were successfully managed with balloon dilation, with over 90% of patients ultimately achieving full oral feeding. Thus, this rate represents the detected burden of a manageable sequela within an effective treatment pathway.

The evolution of risk factors for AS provides compelling evidence for the impact of the surgical learning curve. The initial univariate significance of lower birth weight (16, 17) and higher tension index (8, 16, 18) aligns with established tenets. However, their diminishing significance in the multivariable model suggests (19) that advanced thoracoscopic skill can surmount anatomical adversity. This attenuation is quantitatively reflected in our multivariable model, where birth weight as a continuous variable showed only a minuscule, clinically irrelevant effect (OR 1.001), and the tension index was not retained. These model results, detailed in Supplementary Table S4e, align with the narrative presented in Figure 4, where increasing surgical experience allowed successful management of more challenging, lower birth weight infants without a proportional rise in stricture rates. The evolution of our institutional experience and its impact on overcoming anatomical challenges is graphically summarized in Figure 4.

During the early phase (2017–2019), the team primarily operated on neonates with normal birth weight (76.9%–81.8%) but experienced high AS rates (80.6%–86.7%). As cumulative experience accrued, the team demonstrated an enhanced capability to manage a progressively greater burden of low birth weight (LBW) infants (with the proportion of LBW cases rising from 23.5% to 50%), while the AS rate concurrently exhibited a stabilizing or improving trend (20, 21). This progression underscores that refined techniques effectively neutralized the inherent anatomical disadvantage associated with LBW (22–24). This evolution highlights the importance of the institutional learning curve. In this context, the work of Hyman et al. (25) is highly relevant; they demonstrated that outcomes for thoracoscopic repair are optimized in high-volume, expert centers, supporting the argument for the centralization of complex pediatric surgery to navigate the learning curve and minimize complications such as anastomotic stricture. The transient increase in AS rate in 2024 (85%) likely reflects random variation due to the small sample size (*n* = 17) and a higher proportion of long-gap cases (17, 19, 26), rather than a reversal of progress. This observation directly supports the conclusion that long-gap anatomy is a significant risk factor for anastomotic stricture in our cohort. This apparent paradox—a minute increase in risk with higher birth weight in the multivariable model—is critical to interpret correctly. The effect size (OR 1.001 per 100 g) is vanishingly small and clinically irrelevant. We interpret it as most likely reflecting residual confounding (e.g., by unmeasured technical or healing factors within the adjusted model), not as evidence that heavier infants are at greater risk. This finding in no way undermines the clinical tenet—supported by our own univariate analysis and the literature—that low birth weight poses a greater anatomical and physiological challenge.

The overall AS rate of 74.6% in our cohort must be interpreted within the context of comprehensive long-term surveillance (3, 27–29). This figure aligns with contemporary studies reporting incidences up to 80% when follow-up exceeds three years (15). Our proactive dual-modality surveillance strategy—combining routine esophagography (15, 27, 30) and endoscopy (3, 7, 31, 32)—captured 13.3% of asymptomatic strictures (13, 33) and identified 6.7% of late-presenting strictures (31, 34), thereby providing a more accurate reflection of the true long-term burden, as recommended by structured follow-up guidelines (3, 7, 35). Critically, this high detection rate is clinically meaningful only in the context of effective treatment. We successfully managed all cases of symptomatic anastomotic stricture, ultimately achieving normal oral feeding in over 90% of patients—a core outcome that aligns well with established efficacy benchmarks (3, 7, 35). Our consistent use of x-ray guided balloon dilation (16, 35–37)—endorsed by the ERNICA consensus as a primary management option for anastomotic strictures (3, 8)—enabled this favorable outcome.

In summary regarding the high AS rate: 1) It is amplified by intensive surveillance capturing subclinical disease; 2) It occurs within a context of successful treatment (balloon dilation) leading to excellent functional outcomes; and 3) The statistical modeling indicates that the institutional learning curve has attenuated the impact of traditional patient-related risk factors like low birth weight. Therefore, a high detected rate in this setting is more a testament to comprehensive follow-up and the evolving mastery of a technically demanding procedure than an indicator of poor surgical quality.

Furthermore, in accordance with international guidelines, all patients in our cohort routinely received proton pump inhibitors (PPIs), a practice strongly supported by the ERNICA consensus for antacid prophylaxis (8). While PPIs therapy may mitigate the severity of strictures by reducing acid-induced inflammation (38, 39), it does not entirely prevent their initial development (3, 35, 40–44), as AS formation is often inherent to the anastomotic healing process (45–48). Thus, a high AS rate in this setting is a marker of diligent follow-up and a treatable condition, not a poor ultimate outcome (27, 28).

### 4.4 Recurrent TEF and respiratory anomalies: a distinct etiological pathway

The analysis delineates a distinct etiological pathway for recurrent TEF, culminating in the identification of respiratory system anomalies as its strongest independent predictor (adjusted *OR 8.84, 95% CI 2.09–33.47, p* *=* *0.002*). Notably, this factor was retained for multivariable analysis based on strong clinical plausibility and its significant association in comparative tests (30.8% vs. 4.8%, *p* *=* *0.006*; Table 4), despite its *p*-value in the formal univariate logistic regression screening exceeding our pre-specified threshold—a nuance likely influenced by the low event rate. Its decisive emergence in the multivariable model underscores the critical role of statistical adjustment in isolating its unique effect, powerfully reinforcing the idea that recurrent TEF is less a technical failure and more a consequence of a compromised local tissue environment inherent to specific congenital malformations (3, 14, 25). Anomalies such as severe tracheomalacia or laryngeal clefts can create an operative field prone to inflammation and impaired healing. The overwhelmingly solitary occurrence of recurrent TEF (Figure 3) further corroborates this patient-driven pathogenesis, distinct from technical complications like AL or AS. These findings provide compelling, methodologically robust justification for routine preoperative bronchoscopy, as emphasized by guidelines (3, 13, 47, 48), to identify high-risk patients for tailored surgical planning and informed counseling.

### 4.5 Limitations

This study has several limitations inherent in its single-center, retrospective design. First, despite multivariable adjustment, unmeasured confounding factors and selection bias may still be present. To assess the potential influence of the institutional learning curve, a key source of temporal bias, we conducted a sensitivity analysis by including the “year of surgery” as a covariate in the AS model. Although this did not alter the main conclusions, it highlights that our findings regarding AS are framed within our evolving experience. Second, the definitions of certain variables (e.g., operative complexity) are specific to our center and require external validation. Third, the high baseline incidence of anastomotic stricture (74.6%) in our cohort indicates that “no stricture” was the less common outcome. This not only impacts the statistical power to identify risk factors but also means that our risk model for AS operates in a context where the complication is common rather than rare. Fourth, our analytical approach, while strengthened in this revision, has inherent constraints. The variable selection process for recurrent TEF warrants specific consideration: the strong clinical association of respiratory anomalies did not cross the pre-specified statistical threshold (*p* *<* *0.1*) in the formal univariate logistic regression analysis (Supplementary Table S5), likely due to the low event rate (*n* = 13). Our decision to retain it in the multivariable model was based on strong *a priori* clinical rationale. Its emergence as a robust independent predictor after adjustment underscores the value of combining statistical criteria with clinical reasoning, yet it also highlights the challenges of model building in underpowered analyses. Furthermore, while our sensitivity analyses (Supplementary Table S4) confirmed the robustness of our primary findings to different modeling choices (e.g., treatment of continuous variables), residual confounding cannot be completely excluded. Fifth, the relatively low event rates for anastomotic leak (*n* = 31) and especially recurrent TEF (*n* = 13) limit the statistical power and precision of their respective models; findings for these outcomes should be considered hypothesis-generating. To directly address methodological concerns regarding predictor collinearity and model diagnostics, we performed comprehensive assessments of predictor correlations (Supplementary Table S6) and regression diagnostics (Supplementary Table S7). These analyses support the internal validity and stability of our final models. Finally, the generalizability (external validity) of our results may be restricted. Our findings are from a high-volume center with a specific protocol for intensive surveillance and management. Outcomes may vary in centers with different patient populations, surgical techniques, perioperative care, or follow-up intensity, particularly those in the early stages of their thoracroscopic learning curve.

### 4.6 Conclusion and clinical implementation pathway

Our findings mandate a precision-based framework for mitigating complications in thoracoscopic EA repair. We propose the following pathway:

**AL Prevention:** Preventing AL should focus on optimizing operative efficiency. Our data suggest targeting an operative time of ≤2 h, achievable through standardized protocols and simulation training to enhance technical fluency.

**AS Mitigation:** Mitigating AS requires acknowledging the institutional learning curve. Centralizing care in high-volume centers and implementing proactive, guideline-adherent surveillance can reframe AS as a manageable, expected sequela within a successful treatment pathway.

**Recurrent TEF Surveillance:** Surveillance for recurrent TEF should be prioritized in patients with respiratory anomalies, identified through routine preoperative bronchoscopy. This high-risk subgroup warrants structured postoperative surveillance for early detection.

By aligning specific strategies with the distinct etiology of each complication and adhering to consensus-recommended practices for medication use and stricture management, we can systematically optimize outcomes for these infants.

The internal validity of the risk associations underpinning this framework is supported by the comprehensive methodological rigor applied, including formal variable selection, assessment of predictor correlations, and thorough regression diagnostics (Supplementary Tables S4–S7).

## 5 Conclusion

This study identifies three distinct etiological pathways underlying the major complications following thoracoscopic repair of type III esophageal atresia in a homogeneous neonatal cohort. We demonstrated that anastomotic leak is primarily governed by operative efficiency, best captured by the ≤2-hour operative benchmark (OR 0.24)—a metric reflecting not mere speed, but the proficiency of a coordinated surgical team. Anastomotic stricture (incidence 74.6%) is strongly shaped by the institutional learning curve, while recurrent tracheoesophageal fistula is predominantly associated with inherent respiratory anomalies (OR 8.84). The clear separation in these complication profiles underscores their independent pathophysiology and mandates precision-based strategies.

The robustness of these key associations is supported by comprehensive methodological rigor, including sensitivity analyses (Supplementary Table S4), assessment of predictor correlations (Supplementary Table S6), and regression diagnostics (Supplementary Table S7). To translate these findings into practice, we propose a framework prioritizing operative efficiency for leak prevention, centralized expertise for stricture management, and preoperative bronchoscopy for fistula surveillance. Future multi-center validation is essential to refine these neonatal-specific risk models and develop evidence-based protocols to improve long-term outcomes.

## Data Availability

The data analyzed in this study is subject to the following licenses/restrictions: The datasets are not publicly available because they contain protected health information of the patients. Access to the de-identified datasets is restricted by the Ethics Committee of Guangdong Women and Children Hospital to ensure patient confidentiality and privacy, and is available upon reasonable request subject to ethical review. Requests to access these datasets should be directed to Shangjie Xiao, drsiow@163.com.
