# Supplementary material for: Risk factors for anastomotic complications following thoracoscopic repair of type III esophageal atresia in neonates: a single-center retrospective cohort study
Source: Front Pediatr. 2026 Feb 17;14:1743040. doi: 10.3389/fped.2026.1743040 (PMC12953481; doi:10.3389/fped.2026.1743040)
Supplement: Supplementary file 1 [file Table1.docx]

# Univariate Analysis and Candidate Predictor Screening

## Candidate Predictors Based on Univariate Analysis (p < 0.1)

List of Candidate Predictors (p < 0.1)

| **outcome** | **predictor** | **p_value** | **test_type** |
| --- | --- | --- | --- |
| Anastomotic Leak | Operative_Time_cat | 0.00128955224 | Chi-square |
| Anastomotic Leak | Operative_Time | 0.01033518304 | t-test |
| Anastomotic Leak | Gestational_Age_cat | 0.04140044614 | Chi-square |
| Anastomotic Leak | Birth_Weight_cat | 0.05003928594 | Chi-square |
| Anastomotic Leak | Operation_factor | 0.08633957184 | Chi-square |
| Anastomotic Strictures | Birth_Weight_cat | 0.00223789977 | Chi-square |
| Anastomotic Strictures | Birth_Weight | 0.00307579210 | t-test |
| Anastomotic Strictures | Tension_Index | 0.02393347151 | t-test |
| Anastomotic Strictures | Gestational_Age_numeric | 0.06341819052 | t-test |
| Recurrent TEF | Gastrointestinal_Anomalies | 0.00002618264 | t-test |
| Recurrent TEF | Genitourinary_Anomalies | 0.00140901782 | t-test |
| Recurrent TEF | Respiratory_Anomalies_bin | 0.00193134604 | Chi-square |

## Detailed Univariate Analysis Results - Anastomotic Leak

| **Characteristic** | **No**  N = 170^1^ | **Yes**  N = 31^1^ | **p-value**^2^ |
| --- | --- | --- | --- |
| Age_at_Surgery | 5.19 (3.28) | 6.26 (4.02) | 0.170 |
| Gestational_Age_numeric | 37.81 (2.50) | 38.49 (2.13) | 0.118 |
| Birth_Weight | 2,574.56 (572.80) | 2,723.23 (517.95) | 0.155 |
| Operative_Time | 2.25 (0.71) | 2.80 (1.08) | 0.010 |
| Proximal_Pouch_cat |  |  | 0.754 |
| High (T1-T2) | 69 (40.6%) | 13 (41.9%) |  |
| Low (T3-T4) | 25 (14.7%) | 3 (9.7%) |  |
| Medium (T2-T3) | 76 (44.7%) | 15 (48.4%) |  |
| Preoperative_CT.measured_Gap_Length | 1.28 (0.71) | 1.33 (0.87) | 0.795 |
| Intraoperative_Gap_Length | 1.80 (0.72) | 1.82 (0.85) | 0.928 |
| Tension_Index | 0.75 (0.40) | 0.70 (0.37) | 0.470 |
| Sex_factor |  |  | 0.394 |
| Female | 72 (42.4%) | 10 (32.3%) |  |
| Male | 98 (57.6%) | 21 (67.7%) |  |
| Gestational_Age_cat |  |  | 0.041 |
| Post-term (≥42 weeks) | 0 (0.0%) | 1 (3.2%) |  |
| Preterm (<37 weeks) | 48 (28.2%) | 6 (19.4%) |  |
| Term (37-41 weeks) | 122 (71.8%) | 24 (77.4%) |  |
| Birth_Weight_cat |  |  | 0.050 |
| Extremely low BW (<1500g) | 3 (1.8%) | 1 (3.2%) |  |
| Low BW (1500-2499g) | 66 (38.8%) | 5 (16.1%) |  |
| Normal BW (≥2500g) | 101 (59.4%) | 25 (80.6%) |  |
| Operative_Time_cat |  |  | 0.001 |
| Long (>3h) | 21 (12.4%) | 12 (38.7%) |  |
| Medium (2-3h) | 59 (34.7%) | 8 (25.8%) |  |
| Short (≤2h) | 90 (52.9%) | 11 (35.5%) |  |
| Operation_factor |  |  | 0.086 |
| Basic complexity | 160 (94.1%) | 26 (83.9%) |  |
| High complexity | 6 (3.5%) | 2 (6.5%) |  |
| Moderate complexity | 4 (2.4%) | 3 (9.7%) |  |
| Surgeon_factor |  |  | >0.999 |
| Surgeon 1 | 116 (68.2%) | 21 (67.7%) |  |
| Surgeon 2 | 54 (31.8%) | 10 (32.3%) |  |
| One.Lung_Ventilation_factor | 79 (46.5%) | 11 (35.5%) | 0.350 |
| Any_Anomaly_factor | 64 (37.6%) | 10 (32.3%) | 0.712 |
| Cardiovascular_Anomalies | 18 (10.6%) | 4 (12.9%) | 0.947 |
| Genitourinary_Anomalies | 9 (5.3%) | 1 (3.2%) | 0.970 |
| Gastrointestinal_Anomalies | 14 (8.2%) | 3 (9.7%) | >0.999 |
| Musculoskeletal_Anomalies | 32 (18.8%) | 4 (12.9%) | 0.592 |
| Respiratory_Anomalies_bin | 11 (6.5%) | 2 (6.5%) | >0.999 |
| ^1^Mean (SD); n (%) | | | |
| ^2^Welch Two Sample t-test; Pearson's Chi-squared test | | | |

## Detailed Univariate Analysis Results - Anastomotic Stricture

| **Characteristic** | **No**  N = 51^1^ | **Yes**  N = 150^1^ | **p-value**^2^ |
| --- | --- | --- | --- |
| Age_at_Surgery | 4.84 (2.73) | 5.53 (3.61) | 0.160 |
| Gestational_Age_numeric | 37.27 (2.99) | 38.13 (2.21) | 0.063 |
| Birth_Weight | 2,366.08 (658.67) | 2,676.17 (509.80) | 0.003 |
| Operative_Time | 2.39 (0.78) | 2.32 (0.81) | 0.574 |
| Proximal_Pouch_cat |  |  | 0.337 |
| High (T1-T2) | 23 (45.1%) | 59 (39.3%) |  |
| Low (T3-T4) | 4 (7.8%) | 24 (16.0%) |  |
| Medium (T2-T3) | 24 (47.1%) | 67 (44.7%) |  |
| Preoperative_CT.measured_Gap_Length | 1.36 (0.76) | 1.27 (0.73) | 0.461 |
| Intraoperative_Gap_Length | 1.86 (0.77) | 1.79 (0.73) | 0.534 |
| Tension_Index | 0.86 (0.44) | 0.70 (0.37) | 0.024 |
| Sex_factor |  |  | 0.667 |
| Female | 19 (37.3%) | 63 (42.0%) |  |
| Male | 32 (62.7%) | 87 (58.0%) |  |
| Gestational_Age_cat |  |  | 0.254 |
| Post-term (≥42 weeks) | 0 (0.0%) | 1 (0.7%) |  |
| Preterm (<37 weeks) | 18 (35.3%) | 36 (24.0%) |  |
| Term (37-41 weeks) | 33 (64.7%) | 113 (75.3%) |  |
| Birth_Weight_cat |  |  | 0.002 |
| Extremely low BW (<1500g) | 3 (5.9%) | 1 (0.7%) |  |
| Low BW (1500-2499g) | 25 (49.0%) | 46 (30.7%) |  |
| Normal BW (≥2500g) | 23 (45.1%) | 103 (68.7%) |  |
| Operative_Time_cat |  |  | 0.869 |
| Long (>3h) | 9 (17.6%) | 24 (16.0%) |  |
| Medium (2-3h) | 18 (35.3%) | 49 (32.7%) |  |
| Short (≤2h) | 24 (47.1%) | 77 (51.3%) |  |
| Operation_factor |  |  | 0.557 |
| Basic complexity | 46 (90.2%) | 140 (93.3%) |  |
| High complexity | 2 (3.9%) | 6 (4.0%) |  |
| Moderate complexity | 3 (5.9%) | 4 (2.7%) |  |
| Surgeon_factor |  |  | 0.545 |
| Surgeon 1 | 37 (72.5%) | 100 (66.7%) |  |
| Surgeon 2 | 14 (27.5%) | 50 (33.3%) |  |
| One.Lung_Ventilation_factor | 25 (49.0%) | 65 (43.3%) | 0.587 |
| Any_Anomaly_factor | 22 (43.1%) | 52 (34.7%) | 0.360 |
| Cardiovascular_Anomalies | 9 (17.6%) | 13 (8.7%) | 0.130 |
| Genitourinary_Anomalies | 3 (5.9%) | 7 (4.7%) | >0.999 |
| Gastrointestinal_Anomalies | 6 (11.8%) | 11 (7.3%) | 0.489 |
| Musculoskeletal_Anomalies | 6 (11.8%) | 30 (20.0%) | 0.265 |
| Respiratory_Anomalies_bin | 4 (7.8%) | 9 (6.0%) | 0.894 |
| ^1^Mean (SD); n (%) | | | |
| ^2^Welch Two Sample t-test; Pearson's Chi-squared test | | | |

## TEFDetailed Univariate Analysis Results - Recurrent TEF

| **Characteristic** | **No**  N = 188^1^ | **Yes**  N = 13^1^ | **p-value**^2^ |
| --- | --- | --- | --- |
| Age_at_Surgery | 5.35 (3.46) | 5.46 (2.85) | 0.891 |
| Gestational_Age_numeric | 37.89 (2.51) | 38.24 (1.57) | 0.472 |
| Birth_Weight | 2,592.47 (573.03) | 2,670.00 (465.47) | 0.576 |
| Operative_Time | 2.33 (0.81) | 2.43 (0.68) | 0.616 |
| Proximal_Pouch_cat |  |  | 0.462 |
| High (T1-T2) | 78 (41.5%) | 4 (30.8%) |  |
| Low (T3-T4) | 27 (14.4%) | 1 (7.7%) |  |
| Medium (T2-T3) | 83 (44.1%) | 8 (61.5%) |  |
| Preoperative_CT.measured_Gap_Length | 1.29 (0.75) | 1.23 (0.56) | 0.720 |
| Intraoperative_Gap_Length | 1.81 (0.72) | 1.79 (1.05) | 0.959 |
| Tension_Index | 0.75 (0.39) | 0.73 (0.50) | 0.912 |
| Sex_factor |  |  | 0.200 |
| Female | 74 (39.4%) | 8 (61.5%) |  |
| Male | 114 (60.6%) | 5 (38.5%) |  |
| Gestational_Age_cat |  |  | 0.915 |
| Post-term (≥42 weeks) | 1 (0.5%) | 0 (0.0%) |  |
| Preterm (<37 weeks) | 51 (27.1%) | 3 (23.1%) |  |
| Term (37-41 weeks) | 136 (72.3%) | 10 (76.9%) |  |
| Birth_Weight_cat |  |  | 0.518 |
| Extremely low BW (<1500g) | 4 (2.1%) | 0 (0.0%) |  |
| Low BW (1500-2499g) | 68 (36.2%) | 3 (23.1%) |  |
| Normal BW (≥2500g) | 116 (61.7%) | 10 (76.9%) |  |
| Operative_Time_cat |  |  | 0.647 |
| Long (>3h) | 30 (16.0%) | 3 (23.1%) |  |
| Medium (2-3h) | 62 (33.0%) | 5 (38.5%) |  |
| Short (≤2h) | 96 (51.1%) | 5 (38.5%) |  |
| Operation_factor |  |  | 0.571 |
| Basic complexity | 173 (92.0%) | 13 (100.0%) |  |
| High complexity | 8 (4.3%) | 0 (0.0%) |  |
| Moderate complexity | 7 (3.7%) | 0 (0.0%) |  |
| Surgeon_factor |  |  | 0.402 |
| Surgeon 1 | 130 (69.1%) | 7 (53.8%) |  |
| Surgeon 2 | 58 (30.9%) | 6 (46.2%) |  |
| One.Lung_Ventilation_factor | 85 (45.2%) | 5 (38.5%) | 0.853 |
| Any_Anomaly_factor | 69 (36.7%) | 5 (38.5%) | >0.999 |
| Cardiovascular_Anomalies | 21 (11.2%) | 1 (7.7%) | >0.999 |
| Genitourinary_Anomalies | 10 (5.3%) | 0 (0.0%) | 0.847 |
| Gastrointestinal_Anomalies | 17 (9.0%) | 0 (0.0%) | 0.537 |
| Musculoskeletal_Anomalies | 34 (18.1%) | 2 (15.4%) | >0.999 |
| Respiratory_Anomalies_bin | 9 (4.8%) | 4 (30.8%) | 0.002 |
| ^1^Mean (SD); n (%) | | | |
| ^2^Welch Two Sample t-test; Pearson's Chi-squared test | | | |
