# Supplementary figures and images for: Risk factors for anastomotic complications following thoracoscopic repair of type III esophageal atresia in neonates: a single-center retrospective cohort study
Source: Front Pediatr. 2026 Feb 17;14:1743040. doi: 10.3389/fped.2026.1743040 (PMC12953481; doi:10.3389/fped.2026.1743040)

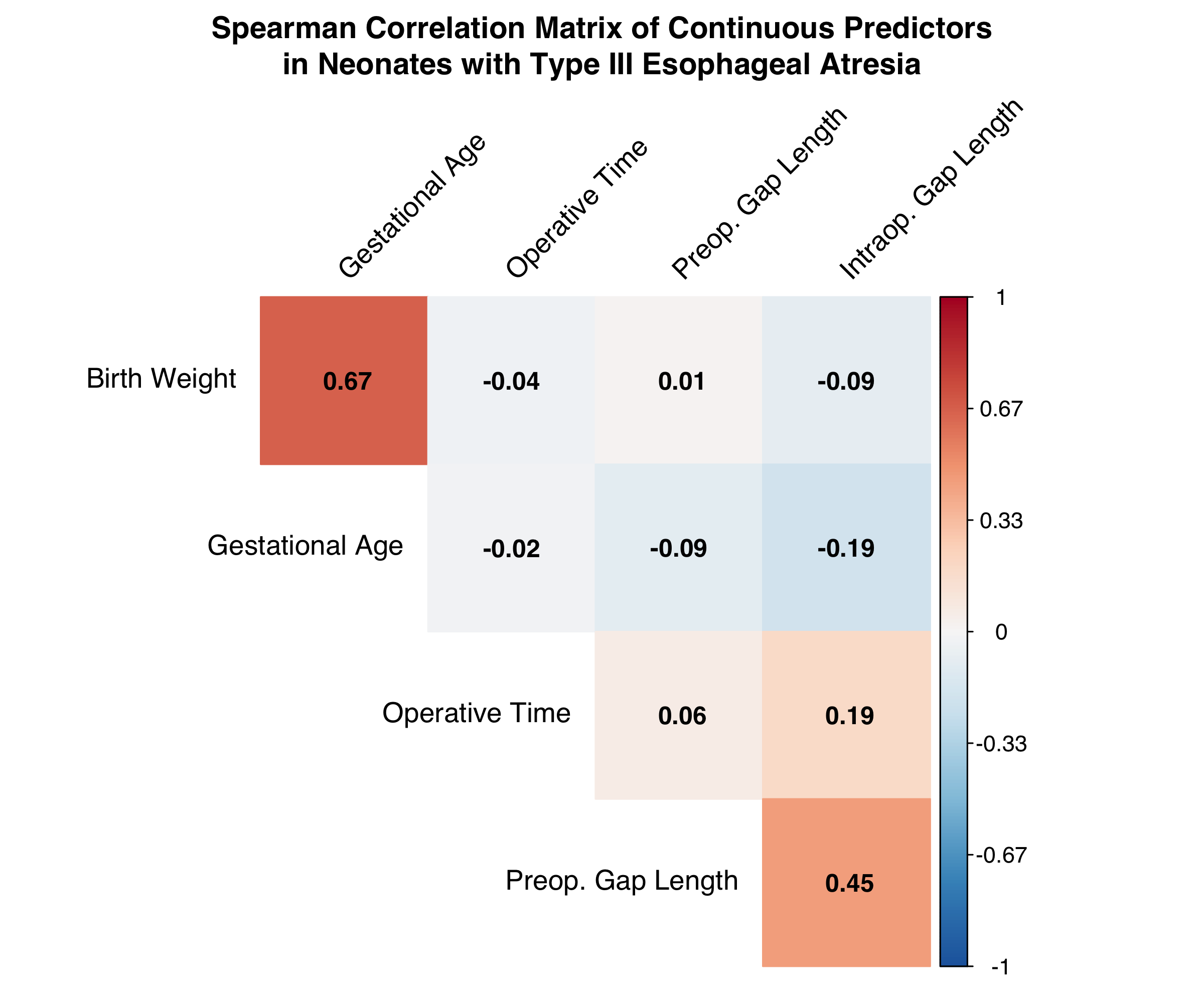

Supplement: Supplementary file 4 [file Image1.png]
